# Inflammation and MiR-21 Pathways Functionally Interact to Downregulate PDCD4 in Colorectal Cancer

**DOI:** 10.1371/journal.pone.0110267

**Published:** 2014-10-13

**Authors:** Oliver Peacock, Andrew C. Lee, Fraser Cameron, Rebecca Tarbox, Natasha Vafadar-Isfahani, Cristina Tufarelli, Jonathan N. Lund

**Affiliations:** Surgery Group, Division of Medical Sciences and Graduate Entry Medicine, School of Medicine, University of Nottingham, Royal Derby Hospital, Derby, United Kingdom; The Ohio State University, United States of America

## Abstract

Inflammation plays a direct role in colorectal cancer (CRC) progression; however the molecular mechanisms responsible for this effect are unclear. The inflammation induced cyclooxygenase 2 (COX-2) enzyme required for the production of Prostaglandin E2 (PGE_2_), can promote colorectal cancer by decreasing expression of the tumour suppressor gene Programmed Cell Death 4 (PDCD4). As PDCD4 is also a direct target of the oncogene microRNA-21 (miR-21) we investigated the relationship between the COX-2 and miR-21 pathways in colorectal cancer progression. Gene expression profile in tumour and paired normal mucosa from 45 CRC patients demonstrated that up-regulation of COX-2 and miR-21 in tumour tissue correlates with worse Dukes' stage. In vitro studies in colonic adenocarcinoma cells revealed that treatment with the selective COX-2 inhibitor NS398 significantly decreased miR-21 levels (p = 0.0067) and increased PDCD4 protein levels (p<0.001), whilst treatment with PGE_2_ up-regulated miR-21 expression (p = 0.019) and down-regulated PDCD4 protein (p<0.05). These findings indicate that miR-21 is a component of the COX-2 inflammation pathway and that this pathway promotes worsening of disease stage in colorectal cancer by inducing accumulation of PGE_2_ and increasing expression of miR-21 with consequent downregulation of the tumour suppressor gene PDCD4.

## Introduction

Colorectal cancer is the third most common cause of cancer related deaths worldwide [1]. Approximately half of all patients diagnosed with colorectal cancer ultimately die of the condition [2]. The five year survival rate has increased to approximately 50–55%, which is attributed mainly to an earlier diagnosis and better tailoring of treatments [3]. Death from colorectal cancer can be prevented by early stage disease detection, but unfortunately it is often detected at an advanced stage when prognosis is worse [4]. The prognosis in colorectal cancer patients is associated with disease stage at the time of diagnosis.

The exact “trigger” for the development of colorectal cancer is still unknown. In 1990, a series of morphological steps known as the normal mucosa-adenoma-adenocarcinoma sequence in colorectal cancer due to genetic alterations was proposed [5]. However, many genetic events lead to the development of sporadic colorectal cancer; no single event occurs in all cancers and therefore no single pattern is applicable to every tumour [6]. Therefore, understanding specific genetic events that occur in colorectal carcinogenesis may have significant implications for diagnosis, prognosis and potentially gene therapy in the future.

There has been a recent resurgence in interest into the causal link between inflammation and cancer. Epidemiological studies have shown that chronic inflammation predisposes individuals to various types of cancer [7]. It is estimated that 15% to 20% of all cancer deaths worldwide are linked with underlying chronic infections and inflammatory responses within such individuals [7]. There is evidence from animal studies and observations in humans that a daily aspirin might be effective in preventing several common cancers [8], [9]. This has been confirmed recently in follow-up studies of patients recruited originally for randomised trials of daily aspirin versus control in the prevention of vascular events [10]–[12]. In these trials, allocation to aspirin resulted in a 40% reduction in cancer deaths from 5 years onwards [11] and a sustained reduction in cancer-related death at 20 year follow-up [10], [12]. Observational studies have also shown that aspirin use is associated with reduced distant metastasis and recurrence in common adenocarcinomas [13]–[15], suggesting that inflammation could play a role in progression as well as in development of cancer.

One of the possible reasons for the observed chemo preventive effects of aspirin in colorectal cancer is its ability to reduce tumour development by inhibition of cyclooxygenase 2 (COX-2) [16]. There is increasing evidence linking the pro-inflammatory enzyme COX-2 with the development and progression of colorectal cancer. COX-2 is induced in colonic epithelium in active inflammatory bowel disease (IBD) [17] and its up-regulation results in elevated levels of prostaglandin (PG), in particular PGE_2_ that is a downstream mediator of COX-2 and promotes many carcinogenic pathways including cellular proliferation, inhibition of apoptosis and angiogenesis [18]. This contributes to the chronic inflammatory process orchestrating a tumour-supporting microenvironment, further linking inflammation with carcinogenesis.

The mechanistic linkage between inflammation and cancer is still not completely clear. Increasing evidence suggests that micro-RNAs (miRNAs) are involved in the regulation of inflammatory processes and are dysregulated in inflammatory conditions [19], including ulcerative colitis [20]. Therefore miRNAs dysregulation represents a potential molecular mechanism for inflammatory pathways to mediate cancer development and progression [21]. In particular, expression levels of miR-21 are increased in active inflammation in ulcerative colitis, which may be associated with the increased risk of cancer development with this condition [22]. Up-regulation of miR-21 has also been reported in other inflamed states including allergic airway inflammation [23], inflammatory skin conditions [19] and *Helicobacter pylori* associated gastric cancer [24]. miR-21 has been recently demonstrated to be a genuine oncogene in pre-B-cell lymphoma [25] and found to be over-expressed in most tumour types [26]. miR-21 is a potent stimulator of tissue and vascular invasion in colorectal cancer and these effects appear in part mediated by its ability to prevent translation of one of the miR-21 target genes, Programmed Cell Death 4 (PDCD4) [27]. More recently, a study has also shown significant down-regulation of PDCD4 in active IBD compared with inactive IBD, which also correlated with up-regulation of miR-21 [28], further supporting the link between inflammation, miR-21 and carcinogenesis.

Our aim was to investigate whether a functional interaction exist between the COX-2 (pro-inflammatory enzyme with increased expression in CRC) and miR-21 (oncogenic miR overexpressed in CRC) pathways leading to downregulation of the tumour suppressor PDCD4 in colorectal cancer not associated with previous chronic inflammatory disease.

## Materials and Methods

### Human tissues

This study received ethical approval from the Derbyshire Research Ethics Committee for collection of colorectal cancer tissue and matched normal mucosa from patients who underwent surgical resection for colorectal cancer between August and December 2010 at the Royal Derby Hospital, Derby, UK.

All patients diagnosed with colorectal cancer were discussed at the Royal Derby Hospital colorectal Multidisciplinary Team Meeting after radiological staging, and those deemed suitable for resection with curative intent were eligible for inclusion. Written informed consent was taken and patients unable or not willing to provide informed consent were excluded from the study, as were those withdrawing consent at any stage.

### Cell Culture

The HCA-7 cell line was obtained from the Health Protection Agency Culture Collection (HPACC, Porton Down, UK). Cells were cultured in Dulbecco's Modified Eagle Media (DMEM) (GIBCO, Paisley, UK) supplemented with 10% foetal bovine serum (Fisher Scientific, Loughborough, UK), 2mM L-Glutamine (Sigma-Aldrich, Poole, UK), penicillin (50 units/ml) and streptomycin (50 µg/ml) (Sigma-Aldrich). Cells were cultured in 75cm^3^ vented flasks (TSZ Scientific, Framingham, MA, USA) in humidified incubators at 37°C with 5% CO_2_ (Sanyo, Osaka, Japan).

### Cell Line Transfections and treatments

Cells were seeded at 500,000 cells/well into a 6 well plate and incubated at 37°C with 5% CO_2_ until they reached 70% confluence.

For miR-21 inhibition studies, cells were transfected with miR-21 inhibitor (100nM) or a negative scrambled control (100nM) (miRIDIAN, Dharmacon Lafayette, CO, USA), using Dharmafect 2 lipid transfection reagent (Dharmacon Lafayette, CO, USA) according to the manufacturer's instructions.

For COX-2 inhibition, cells were treated with serum free medium containing the control (0.1% DMSO) or 100 µM NS398 (selective COX-2 inhibitor, Cayman Chemical, Michigan, USA) for 72 hours. For Prostaglandin E_2_ (PGE_2_) treatment, cells were grown 24 hours with serum free medium containing DMSO vehicle alone or 1 µM PGE_2_ so that the final concentration of DMSO in both conditions was the same (0.1%). For combined miR-21 inhibition and PGE_2_ treatment, PGE_2_ was added to the culture 48 hours after miR-21 inhibitor transfection and cells were cultured for a further 24 hours.

### RNA analyses

Total RNA extraction was performed using TRI Reagent following the manufacturer's protocol (Sigma-Aldrich) and quantified using a Nanodrop spectrophotometer (Thermo Fisher Scientific, Wilmington, DE, USA).

For the miR-21 studies, a total of 6 ng of total RNA was used to reverse transcribe miR-21 and RNU44 control into cDNA following the TaqMan miRNA protocol (Applied Biosystems, Foster City, CA, USA), using hairpin primers directed to miR-21 and RNU44 as a control in a thermocycler (GeneAmp PCR System 9700, Applied Biosystems) for 30min at 16°C, 30 min at 42°C, 5min at 85°C. Real-time quantitative polymerase chain reaction was then performed using miRNAs specific TaqMan probe assays (miR-21, ID 000397; RNU44, ID 001094; Applied Biosystems) in a Chromo4 thermal cycler (Bio-Rad Laboratories LTD, Hemel Hempstead, UK). miR-21 expression levels were normalised to RNU44 and calculated using the 2^−ΔΔCt^ method [29] using commercially available normal colon RNA as a calibrator.

For *COX-2* and *PDCD4* analyses, 30ng of total RNA was converted to cDNA with random hexamers using the TaqMan high capacity cDNA reverse transcription protocol (Applied Biosystems, Foster City, CA, USA). Real time PCRs were performed according to the manufacturer instructions in a Chromo4 real time cycler (Bio-Rad Laboratories Ltd) using commercially available 20x TaqMan assays (Applied Biosystems) for *PDCD4* (Hs00377253_m1) and *COX-2* (PTGS2 - Hs00153133_m1), alongside the control genes *GAPDH* (Hs02258991_g1), *PGK1* (Hs00943178_g1) and *HPRT* (Hs01003267_m1). For the experiments in cell lines, quantification was performed in accordance to MIQE (Minimum Information for Publication of Quantitative Real-Time PCR Experiments) guidelines [30] relatively to the geometric mean of the reference genes *GAPDH*, *PGK1*, *HPRT* using the GenEX software (MultiD Analysis; Göteborg, Sweden). Due to limited sample's amounts, in human tissues expression was quantified relatively to *GAPDH* alone as described above for miR-21 analyses.

### Protein analyses

Protein extractions and quantification were performed as previously described [31]. Western blotting was performed using the loading control anti-beta actin antibody (1∶1000 dilution; Abcam, Cambridge, UK) in combination with anti-PDCD4 antibody (1∶500 dilution; Sigma-Aldrich) or anti-COX-2 (1∶1000 dilution; Abcam). Anti-rabbit IgG antibody conjugated to alkaline phosphatase was used as secondary antibody (1∶30,000 dilution; DAKO, Ely, UK) for detection of primary antibody binding. Immunocomplexes were visualised by enhanced chemiluminescence using the ECL kit (Bio-Rad) according to the manufacturer's protocol. The chemidoc system was used to capture images and Quantity One (Bio-Rad) software was used for quantification of bands' intensities.

### Elisa Analysis

PGE_2_ levels were measured in the media of HCA-7 treated and untreated cells using the PGE_2_ elisa detection kit (Cayman Chemical, Ann Arbor, Michigan USA) according to the manufacturer's instructions. Plates were read with a plate photocytometer at 450 nm (Wallac 1420 Victor; Perkin-Elmer, Waltham, MA, USA).

### Statistical Analysis

Statistical analysis was performed using GraphPad Prism Version 5.03 (GraphPad Software, La Jolla, CA, USA). The Kolmogorov-Smirnov test demonstrated all the tissue sample data to be non-parametric and all the cell line data to be parametric. Therefore patient's data is expressed as medians and range, whilst cell line data is expressed as mean and standard error of the mean. The Wilcoxon signed-rank test (paired data); the Mann-Whitney U test (unpaired) and the Kruskall-Wallis test were used for comparative analysis of patient's data. The paired t-test (paired data); the t-test (unpaired) and the ANOVA test were used for comparative analysis of cell line's data.

Statistical significance was determined at p≤0.05 for both patient's and cell line's data.

## Results

### Increase in miR-21 directly relates to increase in COX-2 mRNA levels in CRC patients

We performed our study on primary colorectal cancer tissue and paired normal mucosa collected between August and December 2010 from 45 elective patients with sporadic cases of CRC. A prospectively maintained database was populated with patient demographics, neoadjuvant therapies and tumour characteristics. The tumour histopathology was classified according to the national minimum data set for colorectal cancer designed by the Royal College of Pathologists, UK [32]. No patients were excluded or had withdrawn from the study and none had benign disease. The median age was 69 (range 51–88) years and the majority were male patients. Four patients with rectal cancer who received neo-adjuvant chemo-radiotherapy were also included based on previous studies demonstrating that expression of miR-21 is equivalent between radiated and non-radiated tissue [33]. All patients had a complete resection and histology confirmed all tumours were adenocarcinomas. (Table S1 and S2 in File S1)

**Figure 1:**
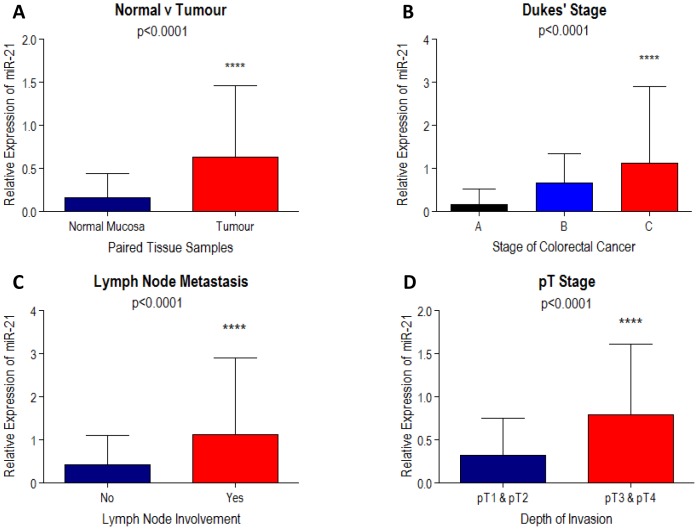
miR-21 expression in primary colorectal tissue samples, calculated relatively to that of the control miRNA RNU44, was studied in primary tumour and matched adjacent normal mucosa (n = 45; A); in Dukes' A (n = 9), B (n = 23) and C (n = 13) stages (B); in lymph node negative (No; n = 32) versus lymph node positive (Yes, n = 13) cases (C); and in tumours displaying lower (pT1 & pT2; n = 11) versus higher (pT3 & pT4; n = 34) depth of invasion (D). Column bar graphs indicate the median and whiskers demonstrate the IQR of miR-21 expression. Note that significant increase in miR-21 expression is observed in tumours, and this increases further with worsening stage, lymphnode involvement and depth of invasion. Statistical significance was calculated using the Wilcoxon signed-rank test in A, the Kruskall-Wallis test in B, and the Mann-Whitney U test in C and D.

Using by real time RT-PCR we studied expression of *COX-2* and miR-21 in tumour tissues as compared to normal mucosa in our cohort of CRC patients. Relative expression of miR-21 was significantly up-regulated in the CRC tissue compared with the matched normal mucosa (p<0.0001, Wilcoxon matched-pairs signed rank test; Fig. 1A). Moreover, expression levels of miR-21 were correlated with the commonly used clinical-pathological features for CRC (Table 1). Higher expression of miR-21 in tumour tissues significantly correlated with a worse Dukes' stage (p<0.0001, Kruskall-Wallis test; Fig. 1B), lymph node metastasis (p<0.0001, Mann-Whitney U test; Fig. 1C), and depth of tumour invasion (pT stage; p<0.0001, Mann-Whitney U test; Fig. 1D). These findings are in agreement with previous reports [34], [35], and confirm that miR-21 is up-regulated in CRC with increasing expression levels correlating with increased severity of the disease.

**Table 1:** Relative expression of miR-21 with common clinical-pathological features of colorectal cancer.

Variables	Patients	p-Value
*Histological Type:*		
Well Differentiated	4	
Moderate & Poorly Differentiated	41	p = 0.0002
*Depth of Invasion:*		
pT1 & pT2	11	p<0.0001
pT3 & pT4	34	
*Dukes' Stage:*		
A	9	
B	23	p<0.0001
C	13	
*Lymph Node Metastasis:*		
Positive	13	p<0.0001
Negative	32	
*Vascular Invasion*		
Positive	13	p = 0.01
Negative	32	

**Figure 2:**
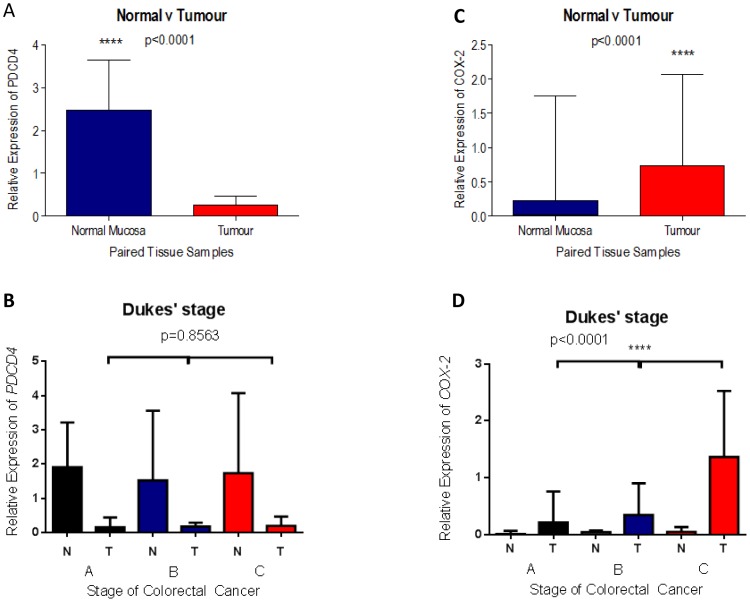
Expression of *PDCD4* and *COX-2* mRNA in primary colorectal tissue samples calculated relatively to that of the control gene *GAPDH*. Combined relative expression of PDCD4 and COX-2 was analysed in primary tumour versus matched adjacent normal mucosa (n = 45; A and C respectively), and in normal (N) and tumour (T) tissues from Dukes' A (n = 9), B (n = 23) and C (n = 13) stages (B and D respectively). The column bar graph indicates the median and the whiskers demonstrate the IQR of mRNA expression. Statistical significance was calculated using the Wilcoxon signed-rank test in A and C; the Kruskall-Wallis test in B and D. Note that *PDCD4* is downregulated in tumour versus normal, but no differences are seen in the remaining expression in tumours of different Dukes' stages. By contrast *COX-2* levels increase in tumour and with disease stage.

Relative expression of *PDCD4* was significantly down-regulated in CRC tissues compared with matched normal mucosa (p<0.0001, Wilcoxon matched-pairs signed rank test; Fig. 2A). However, there was no correlation between *PDCD4* expression in tumour tissues and the Dukes' stage, with expression in Dukes'A stage being already significantly decreased as compared to normal tissue and no further decrease seen in more advanced stages (Fig. 2B). It is unlikely that the observed downregulation of *PDCD4* at the RNA level is caused by miR-21 given that this micro-RNA acts to prevent PDCD4 mRNA translation rather than induce its degradation or transcriptional repression [27]. Given that all the patients studied carried malignant tumours with an assigned Dukes' stage, the data suggest that during progression of malignancy increasing amounts of miR-21 lead to inhibition of translation of the already decreased levels of *PDCD4* mRNA. Ethics constraints prevented us from analysing PDCD4 protein levels in these patients to confirm this hypothesis and we recurred to *in vitro* studies to gain mechanistic insights (see next section).

Interestingly, relative expression of *COX-2* mRNA was significantly up-regulated in tumour tissues compared with their matched normal mucosa (p<0.0001, Wilcoxon matched-pairs signed rank test; Fig. 2C). Moreover, similarly to what was observed with miR-21, relative expression of *COX-2* mRNA in tumour tissues significantly correlated with a worse Dukes' stage (p<0.0001, Kruskall-Wallis test; Fig. 2D). Given that miR-21 and inflammation have both been shown to decrease PDCD4 protein levels [27], [36], a possible interpretation of these findings is that increase in miR-21 and *COX-2* may be functionally related and that they could lead to downregulation of PDCD4 through a common pathway.

### HCA-7 cells are a suitable system for analysing the relationship between miR-21 and COX-2 overexpression in CRC

To further investigate the potential of a functional relationship between COX-2 and miR-21 in the down-regulation of PDCD4 in CRC we recurred to an *in vitro* cell culture system. We chose the human colonic adenocarcinoma cell line HCA-7 isolated from a Dukes' B tumour [37] because it has high endogenous levels of COX-2 [38] and following treatment with the COX-2 inhibitor NS398 increases expression of *PDCD4* [39]. In order to determine whether HCA-7 cells are a good system in which to study the functional relationship between the miR-21 and COX-2 pathways, we first needed to determine whether miR-21 is expressed and is responsible for *PDCD4* downregulation in these cells. To achieve these we used RT-PCR to study the levels of miR-21 in untreated HCA-7 cells or in cells treated with 100nM of a miR-21 inhibitor or scramble control based on published work showing that this concentration is non-cytotoxic and effective in inhibitory studies [40].

**Figure 3:**
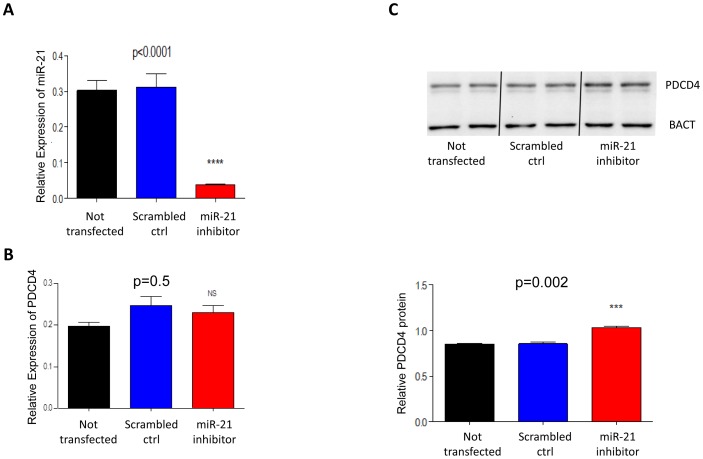
miR-21 and *PDCD4* expression in HCA-7 cells treated with miR-21 inhibitor. Following 72 hours from transfection significant decrease in miR-21 levels are seen only in cells transfected with miR-21 inhibitor (**A**). *PDCD4* mRNA levels were not affected by inhibition of miR-21 (**B**). Western blot analysis of PDCD4 (**C** top panel for a representative blot) reveals a significant increase in protein levels in cells transfected with the inhibitor as compared to untreated or scramble control treated cells, when quantified relatively to beta actin protein (BACT) as a loading control (**C** bottom panel). The column bar graph indicates the mean and the whiskers demonstrate the standard error of the mean (SEM). Statistical significance was calculated using the unpaired t-test. Experiments were repeated three times and analysed in duplicate. Untreated = cells culture in media; scramble = cells transfected with scramble RNA inhibitors; miR-21 inhibitor = cells treated with the miR-21 RNA inhibitor.

We found that miR-21 is expressed at high levels in HCA-7 cells and these levels are not affected by treatment with scramble small RNAs but are significantly decreased (p<0.0001, unpaired t-test) following 72 hours treatment with a specific miR-21 small RNA inhibitor (Fig. 3A). No significant differences in PDCD4 mRNA levels were observed by quantitative RT-PCR in any of the treatment conditions (Fig. 3B); by contrast, a significant increase (p = 0.002, unpaired t-test) in PDCD4 was observed at the protein level by western blot in the miR-21 inhibitor treated cells compared to untreated or scramble control treated cells. These findings are consistent with miR-21 acting to inhibit translation of PDCD4 mRNA rather than directing its degradation and confirm that miR-21 plays a role in PDCD4 downregulation in HCA-7 cells. Therefore both the COX-2 and miR-21 pathways contribute to downregulation of PDCD4 in HCA-7 cells, making them a suitable system in which to study whether a functional relationship exists between the two pathways.

### Relation between miR-21 and COX-2 in the downregulation of PDCD4 in HCA-7 cells

To further investigate the functional interaction between the miR-21 and COX-2 pathways, we first analysed COX-2 mRNA and protein levels in HCA-7 cells after 72 hours treatment with the miR-21 inhibitor compared to scramble control and untreated cells. No difference in COX-2 expression was observed neither at the mRNA nor at the protein levels (p = 0.62 and p = 0.83 respectively, unpaired t test), showing that COX-2 is not a target of miR-21 (Fig. S1 in File S1).

We next treated HCA-7 cells with 100 µM NS398, a specific COX-2 inhibitor reported to be non-cytotoxic and effective at this concentration in cell culture [41], or with vehicle alone (0.1% DMSO). COX-2 plays a critical role during inflammation in the initial steps of the conversion of arachidonic acid to prostaglandins including prostaglandin E (PGE_2_) and up-regulation of COX-2 in colorectal cancer has been shown to associate with marked increase in the production of PGE_2_ [42]. Using Elisa to measure levels of PGE_2_ we found a significant decrease (p = 0.006, unpaired t test) in the media of cells treated with NS398 compared to untreated cells or cells treated with vehicle alone, consistent with inhibition of COX-2 catalytic activity (Fig. S2 in File S1).

**Figure 4:**
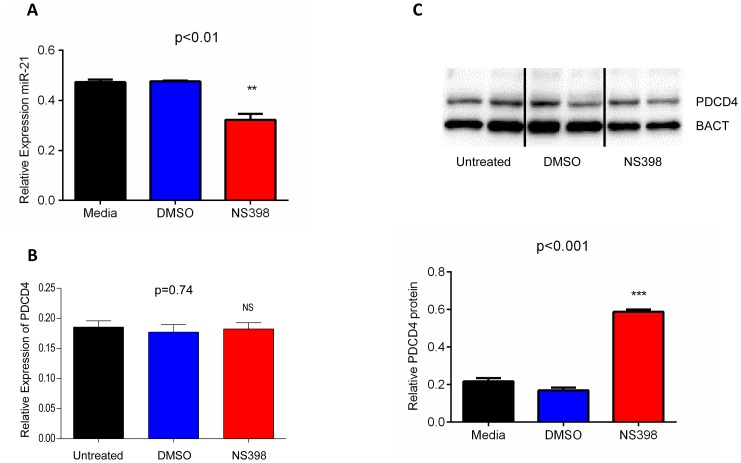
miR-21 and *PDCD4* expression in HCA-7 cells treated with the COX-2 inhibitor NS398. Following 72 hours treatment significant decrease in miR-21 levels is seen in NS398 treated cells (**A**). *PDCD4* mRNA levels are not affected by NS398 treatment (**B**). Western blot analysis of PDCD4 (**C** top panel for a representative blot) reveals a significant increase in protein levels in NS398 treated cells as compared to untreated or vehicle alone (DMSO) treated cells, when quantified relatively to beta actin protein (BACT) as a loading control (**C** bottom panel). The column bar graph indicates the mean and the whiskers demonstrate the standard error of the mean (SEM). Statistical significance was calculated using the unpaired t-test. Experiments were repeated three times and analysed in triplicate. Untreated = cells cultured in media; DMSO = cells cultured in media supplemented with 0.1% DMSO vehicle; NS398 = cells cultured in media supplemented with NS398 prepared in DMSO to a final concentration of 100 µM NS398 and 0.1% DMSO.

Having established effectiveness of the treatment we then measured the relative expression of miR-21 and we could detect a significant decrease in miR-21 following NS398 treatment for 72 hours (p<0.01, unpaired t test; Fig. 4A). By contrast, treatment of HCA-7 cells with NS398 did not alter expression of PDCD4 mRNA (p = 0.74, unpaired t test) (Fig. 4B), an observation that is in contrast to the previously reported 1.5 fold upregulation of PDCD4 mRNA following treatment of HCA-7 cells with NS398 [39]. The difference might be due to the methods used: the latter study analysed PDCD4 mRNA expression by northern blot and quantified it relatively to GAPDH, whilst in our study we have measured mRNA levels using quantitative RT-PCR and normalised the data using the geometric mean of three reference genes (GAPDH, PGK and HPRT) in accordance to the MIQE guidelines [30] (see methods). Our real time PCR data suggests that NS398 treatment leads to a decrease in GAPDH levels (Fig. S3 in File S1) and this change would result in an apparent increase in PDCD4 levels if GAPDH was used as the sole reference gene. Therefore we conclude that NS398 treatment does not alter PDCD4 transcription rate or mRNA stability; however, consistent with the observed decrease in miR-21, significant up-regulation in PDCD4 protein expression (p<0.001, unpaired t test) was observed in NS398 treated cells as compared to untreated or vehicle alone cells (Fig. 4C). These data indicate that down-regulation of PDCD4 by COX-2 is not a consequence of decreased PDCD4 mRNA synthesis or stability, but rather that COX-2 may act by promoting an increase in miR-21 which in turn inhibits translation of PDCD4 mRNA.

### COX-2 activation of miR-21 in HCA-7 cells is mediated by PGE_2_

**Figure 5:**
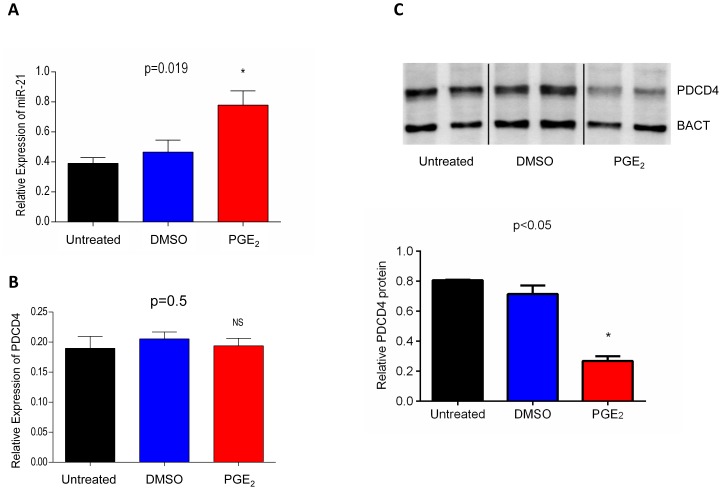
miR-21 and *PDCD4* expression in HCA-7 cells treated with PGE_2_. After 24 hours treatment significant increase in miR-21 levels is seen in PGE_2_ treated cells (**A**). *PDCD4* mRNA levels are not affected by PGE_2_ treatment (**B**). Western blot analysis of PDCD4 (**C** top panel for a representative blot) reveals a significant decrease in protein levels in PGE_2_ treated cells as compared to untreated or vehicle alone (DMSO) treated cells, when quantified relatively to beta actin protein (BACT) as a loading control (**C** bottom panel). The column bar graph indicates the mean and the whiskers demonstrate the standard error of the mean (SEM). Statistical significance was calculated using the unpaired t-test. Experiments were repeated three times and analysed in duplicate. Untreated = cells cultured in media; DMSO = cells cultured in media supplemented with 0.1% DMSO vehicle; PGE_2_ = cells cultured in media supplemented with PGE_2_ prepared in DMSO to a final concentration of 1 µM PGE_2_ and 0.1% DMSO.

Given the role of COX-2 in the production of prostaglandins, to investigate the mechanism underlying activation of miR-21 by COX-2 we treated HCA-7 cells with 1 µM PGE_2_ for 24 hours as this concentration was shown to work effectively on these cells [38]. We found that miR-21 expression was significantly up-regulated (p = 0.019, unpaired t test) in PGE_2_ treated cells as compared to vehicle (0.1% DMSO) treated or untreated cells (Fig. 5A). No changes in PDCD4 mRNA levels were observed in PGE_2_ treated cells (Fig. 5B); however a significant decrease (p<0.05, unpaired t test) in PDCD4 protein levels was observed which paralleled the increase in miR-21 (Fig. 5C). Combined treatment with PGE_2_ and miR-21 inhibitor brought the levels of miR-21 down to levels comparable with those in untreated cells (Fig. S4 in File S1), and treatment with aspirin also led to a decrease in miR-21 levels (Fig. S5 in File S1). These data suggest that COX-2 driven downregulation of PDCD4 in this model system is mainly due to PGE_2_ induced upregulation of miR-21.

## Discussion

Dysregulation of miRNAs is a recognised step in the pathogenesis of many cancer types, including CRC. miR-21 is a miRNA frequently upregulated in CRC, whose increased expression levels are associated with poorer therapeutic outcomes in CRC [34]. We have confirmed that over-expression of miR-21 correlates significantly with worse pathological staging in our CRC patient's cohort (Fig. 1), comprised of patients who presented with tumours of an assigned Dukes' stage, suggesting an important role of miR-21 in cancer invasion and dissemination.

Few of the potential downstream targets regulated by miR-21 have been confirmed, and these include the programmed cell death 4 (PDCD4) tumour suppressor gene that inhibits neoplastic transformation [43]. Recent studies have shown a progressive reduction in PDCD4 expression from normal mucosa, to polyp, to colorectal tumour [33]. Interestingly a significant increase in miR-21 was seen in CRC compared to normal, but not between adenomatous polyps and normal, although a decrease in PDCD4 expression was evident at this transition [33]. In our cohort we observed a decrease in PDCD4 mRNA levels in the tumours relative to the normal tissues, but no changes as disease progressed by Dukes' stage (Fig. 2A). Taken together these observations suggest that at the transition from normal to hyperproliferation downregulation of PDCD4 might be due to regulation at the level of transcription and/or RNA and protein stability. However, in the transition to malignancy miR-21 mediated inhibition of translation of PDCD4 protein levels of the already decreased levels of *PDCD4* mRNA becomes predominant, thus promoting further progression of malignancy. Therefore determining the upstream factors that influence miR-21 expression is instrumental in establishing whether and how miR-21 contributes to malignancy progression and to provide potential therapeutic targets.

Given that in our cohort of CRC patients upregulation of miR-21 is positively related to that of COX-2 mRNA and with worse pathological staging (Fig. 1 and 2B), we hypothesised that miR-21 upregulation in CRC might be linked to an inflammatory response. Circumstantial evidence supporting this hypothesis includes the observations that inflammation can lead to down-regulation of PDCD4 in colorectal tissue [36] and that miR-21 upregulation in gastric cancer is due to PGE_2_ induced activation of NF-kB [44]. Using an *in vitro* culture system we have shown that in HCA-7 cells PDCD4 downregulation is mediated by miR-21 at the post-transcriptional level as no change in PDCD4 mRNA levels were seen (Fig. 3). This confirms the work of a previous group who identified a conserved target site for miR-21 within the 3′-UTR of PDCD4 [27]. We found that whilst down-regulation of miR-21 by transfection of a miR-21 inhibitor had no effect on the expression of COX-2 (Fig. S1 in File S1), inhibition of COX-2 by the specific COX-2 inhibitor NS398 or use of the anti-inflammatory aspirin led to decrease in miR-21 levels (Fig. 3 and Fig. S5 in File S1). In NS398 treated cells, miR-21 downregulation correlates with an increase in PDCD4 protein without changes in PDCD4 mRNA levels (Fig. 4), further supporting a role of miR-21 in the downregulation of PDCD4. Previous studies have demonstrated that NS398 exerts several anti-carcinogenic effects in colon cancer by inducing apoptosis [45], whilst also inhibiting cell cycle progression [39], angiogenesis [46] and metastasis [47]. Our novel finding of a significant up-regulation in PDCD4 protein levels following NS398 treatment suggests that the chemo preventive effects of NS398 may in part be attributed to its action on PDCD4. Moreover, the relationship between COX-2 and miR-21 is unidirectional, whereby increase in COX-2 activity drives overexpression of miR-21; therefore miR-21 is a component of the COX-2 pathway, downstream of COX-2. Indeed we found that miR-21 upregulation is a consequence of COX-2 mediated PGE_2_ production as treatment of cells with PGE_2_ led to increase in miR-21 and decrease in PDCD4 protein, which was reversed by combining PGE_2_ and miR-21 inhibitor treatments (Fig. 5 and Fig. S2 in File S1). These findings are in agreement with work by other groups indicating that COX-2 inhibitors induce apoptosis and inhibit angiogenesis *in vitro*, [48], [49] whilst also attenuating invasion, angiogenesis and metastasis *in vivo* [50], [51].

**Figure 6:**
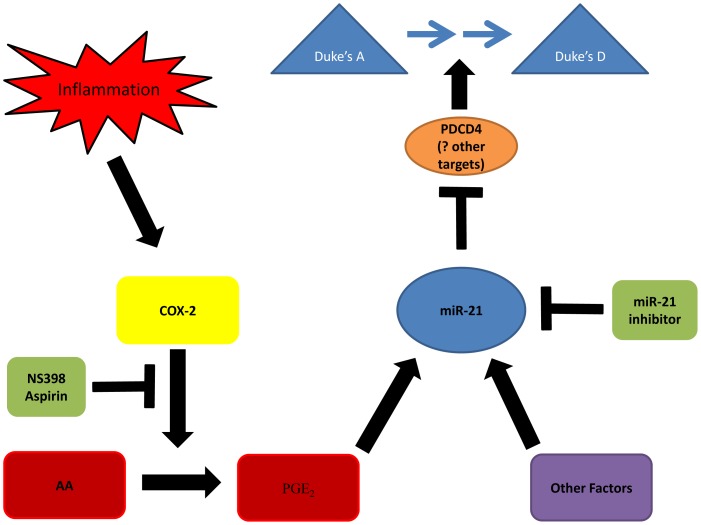
Proposed model of how the inflammation pathway can promote colorectal cancer progression by linking inflammation to downregulation of PDCD4 via miR-21 induction mediated by COX-2 driven accumulation of PGE_2_. Persistent inflammation at the tumour site drives increase in COX-2 expression leading to accumulation of PGE_2_. This in turn leads to the increase in expression of miR-21 and subsequent downregulation of PDCD4 protein levels facilitating progressive increase to more invasive and metastatic forms of colorectal cancer (from Dukes' A to B, C and D). Our experiments indicate that this pathway can be blocked by COX-2 inhibitors (NS398 and Aspirin) by preventing accumulation of PGE_2_ as well as by miR-21 inhibitors. ↑ Activation, 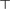 Inhibition. See text for further details.

Our study provides mechanistic insights into the link between inflammation, miR-21 and PDCD4 expression and colorectal cancer progression. Based on our findings it is feasible to propose a model of one of the ways in which persistent inflammation at a site of invasive early stage colorectal tumour can induce progression to more malignant states (Fig. 6). According to our model, continued inflammation at the tumour site leads to progressive increase in COX-2 expression and consequent PGE_2_ accumulation which can be blocked by the use of NS398 COX-2 inhibitor (Fig. 6, left side). PGE_2_ induces increase in miR-21 expression causing further downregulation of PDCD4 protein levels (and potentially other miR-21 targets) thus facilitating further progression of the tumour to more malignant stages and these effects can be reversed using miR-21 inhibitors (Fig. 6, right side). Interestingly, it has just been reported that the 15-hydroxyprostaglandin dehydrogenase (15-PGDH) mRNA which codes for a key enzyme required for PGE_2_ degradation, is a direct target of miR-21 in cholangiocarcinoma cells [52]. Although the presence of this miR-21 mediated effect remains to be proven in CRC, the observation suggests that a reinforcing positive feedback may exists whereby increase in miR-21 induced by PGE_2_ favours further accumulation of PGE_2_ by preventing its degradation.

## Conclusions

In conclusion, we have highlighted the potential benefits of analysing COX-2 and miR-21 expression levels for tumour staging and of correlating these levels to therapeutic response or disease outcome. Moreover, this study has contributed to an improved understanding of the role of miR-21, PDCD4 and COX-2 in colorectal cancer progression. We have demonstrated a functional link between COX-2, miR-21 and PDCD4, which provides further understanding into the beneficial effects of COX-2 inhibitors in colorectal cancer control. Further investigation of the pathway linking inflammation to miR-21 will reveal key intermediate players and their potential to be targeted therapeutically, with the final goal to determine the feasibility of combining the use of anti-inflammatory drugs, with miR-21 inhibitors and strategies targeting intermediate components in the treatment of CRC. These findings therefore provide a basis for identification of potential therapeutic targets in the future management of colorectal cancer.

## Supporting Information

File S1 **Table of Contents:** **Method S1.** Aspirin treatment of HCA-7 cells (page 1). **Figure S1.** *COX-2* mRNA expression in HCA-7 cells treated with miR-21 inhibitor (page 2). **Figure S2.** PGE2 levels in the media of cells treated with the COX-2 inhibitor NS398 (page 3). **Figure S3.** Expression of *GAPDH* in cells treated with NS398 (page 4). **Figure S4.** miR-21 expression in cells treated with PGE2 and miR-21 inhibitor (page 5). **Figure S5.** miR-21 expression in cells treated with Aspirin (page 6). **Table S1.** Demographics for the cohort of colorectal cancer patients (page 7). **Table S2.** Clinical-pathological staging (page 8).
(PDF)
