# Supplementary material for: Inflammation and MiR-21 Pathways Functionally Interact to Downregulate PDCD4 in Colorectal Cancer
Source: PLoS One. 2014 Oct 13;9(10):e110267. doi: 10.1371/journal.pone.0110267 (PMC4195713; doi:10.1371/journal.pone.0110267)
Supplement: File S1 — Table of Contents: Method S1. Aspirin treatment of HCA-7 cells (page 1). Figure S1. COX-2 mRNA expression in HCA-7 cells treated with miR-21 inhibitor (page 2). Figure S2. PGE2 levels in the media of cells treated with the COX-2 inhibitor NS398 (page 3). Figure S3. Expression of GAPDH in cells treated with NS398 (page 4). Figure S4. miR-21 expression in cells treated with PGE2 and miR-21 inhibitor (page 5). Figure S5. miR-21 expression in cells treated with Aspirin (page 6). Table S1. Demographics for the cohort of colorectal cancer patients (page 7). Table S2. Clinical-pathological staging (page 8). (PDF) [file pone.0110267.s001.pdf]

Contents

Supporting Method S1 - Aspirin treatment of HCA-7 cells 1
Supporting Figure S1 - *COX-2* mRNA expression in HCA-7 cells treated with miR-21 inhibitor........ 2
Supporting Figure S2 - PGE_2 levels in the media of cells treated with the COX-2 inhibitor NS398........ 3
Supporting Figure S3 - Expression of *GAPDH* in cells treated with NS398 4
Supporting Figure S4 - miR-21 expression in cells treated with PGE_2 and miR-21 inhibitor 5
Supporting Figure S5 - miR-21 expression in cells treated with Aspirin........ 6
Supporting Table S1 - Demographics for the cohort of colorectal cancer patients 7
Supporting Table S2 - Clinical-pathological staging........ 8

Supporting Method S1 - Aspirin treatment of HCA-7 cells

Aspirin (Acetylsalicylic acid, A6810 Sigma, UK) was dissolved in EtOH (100%) to a final concentration of 100mg/ml (500mM). A further 1:5 dilution to 20mg/ml (100mM) was performed in culture media, the pH adjusted to 7 with NaOH and the solution filter sterilised through 0.2μm filter prior to addition to the cells. HCA-7 cells were seeded at 500,000 cells/well into a 6 well plate and incubated at 37°C with 5% CO2 overnight or until they reached 70% confluence. Cells were then cultured for an additional 72 hours without any treatment of after the addition of the aspirin solution to a final concentration of 5mM aspirin and 0.01% EtOH. A vehicle alone (0.01% EtOH) was also included to exclude effects due to the solvent used. The concentration of 5mM aspirin was chosen based on published work in which this concentration was found to be non-toxic and effective in cell cultures.[1]

References

1. Bergman M, Djaldetti M, Salman H, Bessler H. Inflammation and colorectal cancer: does aspirin affect the interaction between cancer and immune cells? *Inflammation* 2011;34(1):22-8.

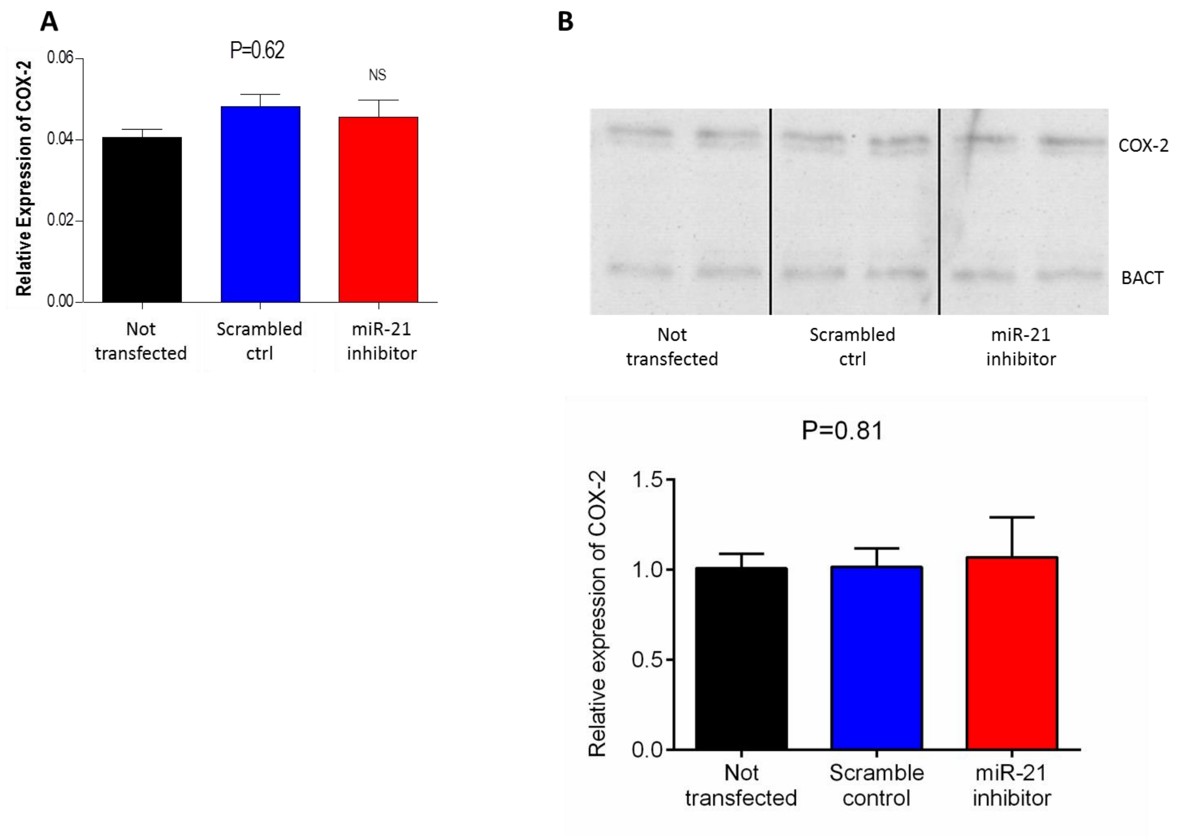

Supporting Figure S1 - *COX-2* mRNA expression in HCA-7 cells treated with miR-21 inhibitor

Following 72 hours from transfection no changes in *COX-2* levels are seen in any of the treatment groups (**A**). Western blot analysis of COX-2 (**B** top panel) also reveals no changes in protein levels in cells transfected with the inhibitor as compared to untreated or scramble control treated cells, when quantified relatively to beta actin protein (BACT) as a loading control (**B** bottom panel). The column bar graph indicates the mean and the whiskers demonstrate the standard error of the mean (SEM). Statistical significance was calculated using the unpaired t-test. Experiments were repeated three times and analysed in duplicate. Not transfected=mock transfected cells; scrambled ctrl =cells transfected with scramble RNA as controls; miR-21 inhibitor=cells treated with the miR-21 RNA inhibitor.

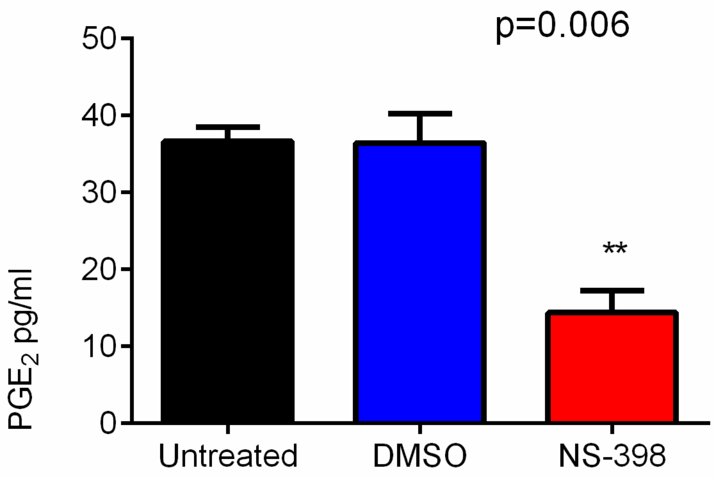

Supporting Figure S2 - PGE_2 levels in the media of cells treated with the COX-2 inhibitor NS398

Following 72 hours treatment significant decrease in PGE2 levels was observed in the media of cells treated with NS398 by Elisa. The column bar graph indicates the mean and the whiskers demonstrate the standard error of the mean (SEM). Statistical significance was calculated using the unpaired t-test. Experiments were repeated three times and analysed in triplicate. Untreated=cells cultured in media; DMSO=cells cultured in media supplemented with 0.1% DMSO vehicle; NS398=cells cultured in media supplemented with NS398 prepared in DMSO to a final concentration of 100mM NS398 and 0.1% DMSO.

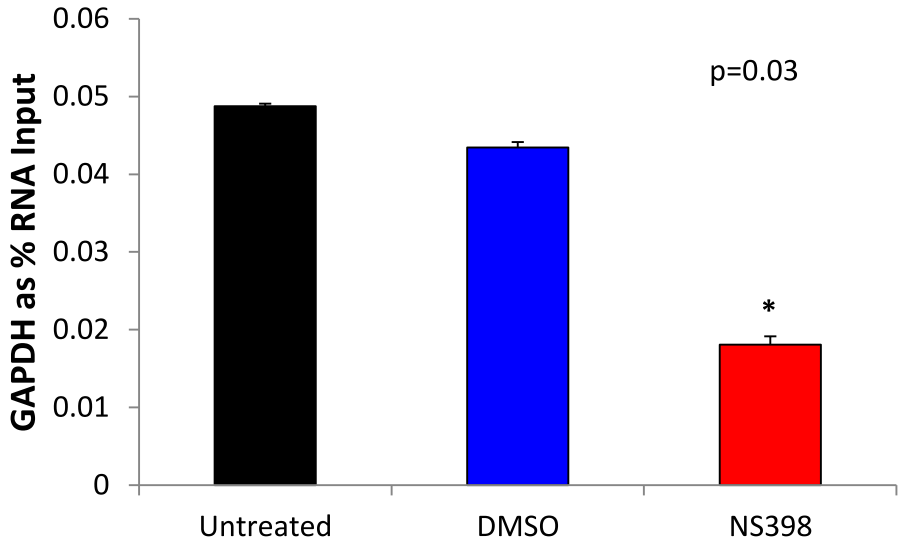

Supporting Figure S3 - Expression of *GAPDH* in cells treated with NS398

Expression is presented as a percentage of RNA Input. A decrease in GAPDH levels was observed in NS398 treated cells relatively to untreated or vehicle alone (0.1% DMSO) treated cells. This suggest that the previously reported 1.5 fold increase in PDCD4 mRNA levels following NS398 treatment of HCA-7 cells [39] might have resulted from using GAPDH as a reference gene. The column bar graph indicates the mean and the whiskers demonstrate the standard error of the mean (SEM). Statistical significance was calculated using the unpaired t-test.

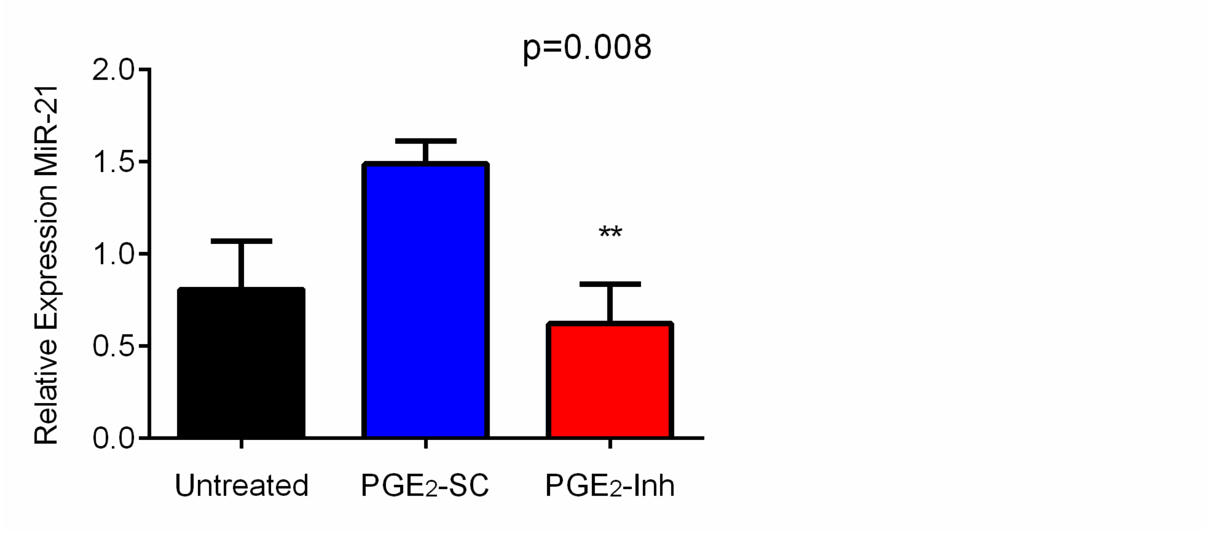

Supporting Figure S4 - miR-21 expression in cells treated with PGE_2 and miR-21 inhibitor

Increase in miR-21 was observed in cells treated with PGE_2 and scramble RNA controls (PGE_2-SC) consistent with what observed in PGE_2 alone treatment (Figure 5). A decrease was observed after addition of PGE_2 to cells transfected with miR-21 inhibitor (PGE_2-Inh). The column bar graph indicates the mean and the whiskers demonstrate the standard error of the mean (SEM). Statistical significance was calculated using the unpaired t-test. Experiments were repeated three times and analysed in duplicate. Untreated=cells cultured in media; PGE_2-SC=cells transfected with scramble RNA as controls and treated with PGE_2; PGE_2-Inh=cells transfected with the miR-21 RNA inhibitor and treated with $PGE_{2.}$

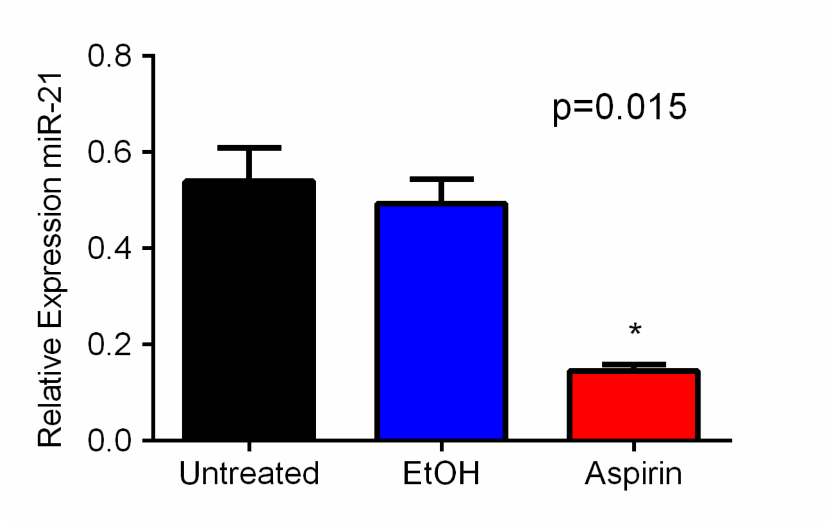

Supporting Figure S5 - miR-21 expression in cells treated with Aspirin

Significant decrease in miR-21 was observed in cells treated with 5mM Aspirin for 72 hours as described in Supporting Methods. The column bar graph indicates the mean and the whiskers demonstrate the standard error of the mean (SEM). Statistical significance was calculated using the unpaired t-test. Experiments were repeated three times and analysed in triplicate. Untreated=cells cultured in media; EtOH=cells cultured in media supplemented with 0.01% ethanol vehicle; Aspirin=cells cultured in media supplemented with Aspirin prepared in ethanol to a final concentration of 5mM Aspirin and 0.01% EtOH.

Supporting Table S1 - Demographics for the cohort of colorectal cancer patients

**Demographics**	**Patients**
Median Age (Range)	69 (51-88)
Patients<br>Male (%)<br>Female (%)	45<br>35 (78%)<br>10 (22%)
Neoadjuvant Therapy	4 (8%)
Adenocarcinoma Histology	45 (100%)
Anastomotic Recurrence	1

Supporting Table S2 - Clinical-pathological staging

Clinical-pathological staging of the specimens using the national minimum data set for colorectal cancer designed by the Royal College of Pathologists

Characteristics	Number of patients
Location of Tumour:	
Colon	29
Rectum	16
Tumour Size:	
<50mm	31
≥50mm	14
Histological Type:	
Well	4
Moderate	38
Poor	3
Pathological Tumour Classification	
pT1	2
pT2	9
pT3	27
pT4	7
Pathological Node Classification	
pN0	32
pN1	9
pN2	4
Pathological Metastasis Classification	
pM0	45
pM1	0
Dukes' Classification	
A	9
B	23
C	13
D	0
Apical Node Involved	
Yes	1
No	44
Lymph Node Ratio	
0-≤0.1	35
0.1-≤0.25	7
0.25-≤0.5	3
>0.5	0
Perineural Invasion	
Yes	4
No	41
Vascular Invasion	
Yes	13
No	32

Lymphocytic Response	
Mild	27
Moderate	17
Severe	1
Complete Resection	
Yes	45
No	0
